# Integrated computational and experimental identification of *N-[(1H-1,2,4-triazol-3-yl)phenyl]-1-(1H-pyrazolo[3,4-b]pyridin-3-yl)methanamide* as a potent and selective TIM-3 inhibitor for NSCLC immunotherapy

**DOI:** 10.3389/fchem.2025.1622511

**Published:** 2025-06-09

**Authors:** Xinxin Ying, Yangyong Lou, Yueming Wu, Weiwei Hu

**Affiliations:** Department of Thoracic Surgery, Affiliated Dongyang Hospital, Wenzhou Medical University, Dongyang, China

**Keywords:** TIM-3, NSCLC, HIT104310526, survival analysis, molecular dynamics, experimental validation

## Abstract

Non-small cell lung cancer (NSCLC) remains a significant clinical challenge, necessitating exploration of novel therapeutic targets such as TIM-3. In this study, integrated computational and experimental methods were utilized to identify potent TIM-3 inhibitors. Survival analysis revealed a significant correlation between elevated TIM-3 expression and decreased patient survival. Structure-based virtual screening and molecular dynamics simulations identified HIT104310526 *(N-[(1H-1,2,4-triazol-3-yl)phenyl]-1-(1H-pyrazolo[3,4-b]pyridin-3-yl)methanamide)*, a candidate exhibiting superior binding affinity and stable interactions within the TIM-3 binding pocket. MMGBSA binding free energy calculations and metadynamics further confirmed its potent binding. Physicochemical evaluations indicated favorable drug-likeness, although solubility improvement is needed. Experimental validation showed selective cytotoxicity of HIT104310526 toward NSCLC cells (A549; IC_50_ = 37.74 μM), with negligible toxicity to normal bronchial epithelial cells (BEAS-2). However, potential cardiotoxicity risks were identified. Collectively, HIT104310526 demonstrates substantial promise as a selective TIM-3 inhibitor, warranting further optimization for NSCLC treatment.

## 1 Introduction

Non-small cell lung cancer (NSCLC) remains one of the most prevalent and lethal malignancies worldwide, with limited efficacy from traditional therapeutic approaches such as chemotherapy and radiation (Alduais et al., 2023). While modern targeted radiotherapy using radiopharmaceuticals has been developed in recent years for NSCLC, exploring other therapeutic strategies remains necessary (Zhu et al., 2023; Klika et al., 2024; Makarem et al., 2025). The advent of immunotherapy, particularly immune checkpoint inhibitors targeting PD-1 and CTLA-4, has significantly improved the prognosis for certain patients (Kong et al., 2024). However, primary or acquired resistance and disease progression continue to challenge clinical outcomes, emphasizing the need to explore additional therapeutic targets (Sharma et al., 2017).

T-cell immunoglobulin and mucin domain-containing protein 3 (TIM-3) has emerged as a promising immune checkpoint molecule involved in immune tolerance and exhaustion, expressed across multiple immune cell types, including CD4^+^ and CD8^+^ T cells, dendritic cells (DCs), natural killer (NK) cells, and myeloid-derived suppressor cells (MDSCs) (Chen et al., 2023). Elevated TIM-3 expression correlates with impaired T-cell function, immune evasion, and poor prognosis across various cancers, including NSCLC (Lei et al., 2020). TIM-3 interacts with multiple ligands, such as Galectin-9 (Yang et al., 2021), CEACAM1(Huang et al., 2015), phosphatidylserine (PtdSer) (DeKruyff et al., 2010), and HMGB1 (Tang and Lotze, 2012), promoting tumor immune tolerance and CD8^+^ T-cell exhaustion, thus facilitating tumor progression (Dixon et al., 2021).

Recent studies highlight that TIM-3 is highly expressed in NSCLC tumor tissues and significantly associated with nodal metastasis, advanced disease stages, and reduced overall survival, positioning TIM-3 as an attractive therapeutic target (Gao et al., 2012; Sauer et al., 2023). Genetic ablation or pharmacological inhibition of TIM-3 has demonstrated promising preclinical results, significantly enhancing anti-tumor immune responses and attenuating tumor progression (Görgülü et al., 2021). Specifically, TIM-3 ablation has been shown to potentiate dendritic cell function through enhanced inflammasome activation and increased oxidative stress, leading to improved CD8^+^ T-cell cytotoxicity and expansion of stem-like memory precursor cells (Dixon et al., 2021). This mechanism provides a robust rationale for targeting TIM-3 in combination with existing checkpoint inhibitors to overcome immune checkpoint resistance and amplify therapeutic outcomes.

Computational drug design offers an efficient approach to accelerate the identification and optimization of novel inhibitors targeting TIM-3, leveraging structure-based virtual screening and dynamic simulation techniques to identify high-affinity compounds (Sadybekov and Katritch, 2023). By integrating bioinformatics and computer-aided drug design methodologies, novel TIM-3-targeted inhibitors can be efficiently screened, optimized, and validated, potentially offering significant clinical benefits in NSCLC treatment (Zhang et al., 2025).

In this study, we aim to identify and characterize TIM-3-targeted inhibitors through advanced computational techniques to enhance immunotherapeutic strategies for NSCLC. The outcomes of this research will provide valuable insights into the design of effective therapeutic agents, potentially overcoming limitations of current immunotherapies and improving clinical management and prognosis of NSCLC patients.

## 2 Methods

### 2.1 Preparation of TIM-3 structure

The crystal structure of TIM-3 in complex with *N-(4-(8-chloro-2-methyl-5-oxo-5,6-dihydro-[1,2,4]triazolo[1,5-c]quinazolin-9-yl)-3-methylphenyl)-1H-imidazole-2-sulfonamide* (compound 38), retrieved from the Protein Data Bank (PDB ID: 7M41) (Rietz et al., 2021), was determined at a resolution of 1.79 Å. Protein preparation was performed using the Protein Preparation Wizard in Maestro (Schrödinger, LLC, New York, NY, 2025), which involved the addition of hydrogen atoms, assignment of bond orders, optimization of hydrogen bonding networks, adjustment of protonation states at physiological pH, and capping of terminal residues. Missing side chains and loops were rebuilt using Prime (Schrödinger, LLC, New York, NY, 2025). The system was subsequently subjected to restrained energy minimization using the OPLS5 force field (Damm et al., 2024), with heavy atom convergence set to 0.3 Å to eliminate steric clashes and unfavorable geometries.

### 2.2 Compound library preparation

A compound library comprising approximately 1.64 million small molecules was obtained from the freely accessible TargetMol database (https://www.targetmol.cn/). Ligand structures were prepared using the LigPrep module in Schrödinger (Schrödinger, LLC, New York, NY, 2025), which included ionization state prediction at pH 7.0 ± 2.0, tautomer generation, and retention of specified chiralities. The geometry of ligands was further optimized using the OPLS5 force field to ensure energy-minimized and stereochemically accurate conformations.

### 2.3 Structure-based virtual screening

To define the binding site, the Receptor Grid Generation panel in Maestro (Schrödinger, LLC, New York, NY, 2025) was used to generate a docking grid centered on the co-crystallized ligand, with a grid box size of 20 Å. Virtual screening was conducted using the Virtual Screening Workflow (VSW) module (Schrödinger, LLC, New York, NY, 2025), integrating LigPrep-prepared ligands and the previously defined receptor grid. Ligands pre-screened via Phase-based pharmacophore modeling were initially subjected to standard precision (SP) flexible docking. The top 10% of ligands, ranked by Glide Score, were further refined using extra precision (XP) flexible docking. Prime/MM-GBSA binding free energy calculations were then performed on these top-scoring XP poses using the default settings in the Prime module (Schrödinger, LLC, New York, NY, 2025), to estimate binding affinity of each protein–ligand complex.

To evaluate whether the identified binding site corresponded to the most energetically favorable region, global docking was performed. Receptor grids covering the entire surface of TIM-3 were generated to allow ligands to explore all possible binding pockets without bias. The same Glide XP docking protocol was applied in this global search. Comparative analysis of docking scores and binding poses between site-specific and global docking verified that the reference compound’s binding region represented the optimal binding pocket. This approach ensured that the selected candidates preferentially targeted the biologically relevant binding site.

### 2.4 ADME and toxicity properties of selective compounds

The ADMET profiles of the selected compounds were evaluated using the ADMETlab 3.0 web server (https://admetmesh.scbdd.com/) (Fu et al., 2024). Parameters related to absorption and distribution included blood–brain barrier (BBB) permeability, human intestinal absorption (HIA), Caco-2 cell permeability, P-glycoprotein (P-gp) substrate classification, and predicted subcellular localization. For metabolic evaluation, interaction probabilities with six major cytochrome P450 isoforms—CYP1A1, CYP1A2, CYP2C9, CYP2D6, CYP2C19, and CYP3A4—were assessed. Toxicity endpoints included AMES mutagenicity, carcinogenic potential, acute oral toxicity, fish toxicity (median lethal concentration, pLC_50_, in mg/L), and rat acute toxicity (lethal dose, LD_50_, in mol/kg).

### 2.5 Binding pose metadynamics (BPMD) simulations

Binding pose stability was further assessed using binding pose metadynamics (BPMD) simulations implemented in the Desmond module of Schrödinger Suite 2025-1 (Schrödinger, LLC, New York, NY, 2025). Each BPMD run was initiated from the docked protein–ligand complex and executed under NPT conditions at 300 K and 1 atm, using the Nosé–Hoover chain thermostat (Beckedahl et al., 2016) and Martyna–Tobias–Klein barostat (Sun et al., 2021). The SPC water model (Mark and Nilsson, 2001) was employed, and the system was solvated with a 10 Å buffer. A harmonic bias potential was applied to the center of mass of the ligand to explore its movement away from the original binding pose.

For each protein–ligand complex, ten independent BPMD simulations were conducted, each lasting 10 ns, with a biasing force constant of 0.2 kcal/mol/Å^2^. PoseScore and PersScore were calculated automatically to evaluate pose stability, where lower PoseScore (<2 Å) and higher PersScore (>0.6) values indicated stable binding (Xu et al., 2024). All simulations used the OPLS5 force field, and default BPMD parameters were applied unless otherwise stated.

### 2.6 All-atom molecular dynamics (MD) simulation

All-atom molecular dynamics (MD) simulations were performed using the Desmond module of Schrödinger Suite 2025-1 within Maestro (Schrödinger, LLC, New York, NY, 2025). Protein–ligand complexes from docking were solvated in a cubic water box with a 10 Å buffer using the SPC model, and 0.15 M NaCl was added to mimic physiological ionic strength. The system was neutralized, and long-range electrostatics were handled using the particle-mesh Ewald method, with a 9.0 Å cutoff applied to both electrostatic and van der Waals interactions.

System relaxation followed Desmond’s default multi-stage equilibration protocol, including Brownian dynamics at 10 K under the NVT ensemble and subsequent restrained and unrestrained equilibration in NPT conditions. Production simulations were conducted under the NPT ensemble using the OPLS5 force field for a total of 1 μs. Temperature and pressure were maintained at 300 K and 1 atm using the Nosé–Hoover chain thermostat and Martyna–Tobias–Klein barostat.

### 2.7 Cell culture

A549 (human lung adenocarcinoma epithelial) and BEAS-2B (human bronchial epithelial) cell lines were purchased from iCell Bioscience Inc. (Shanghai, China). Both cell lines were authenticated via short tandem repeat (STR) profiling. A549 cells were cultured in Ham’s F-12K (Kaighn’s) medium, and BEAS-2B cells in Dulbecco’s Modified Eagle Medium (DMEM). All media were supplemented with 10% exosome-depleted fetal bovine serum (EXO-FBS-50A-1; System Biosciences, Palo Alto, CA, United States) and 1% penicillin-streptomycin (Tianhang Biotechnology, Hangzhou, China). Cells were maintained at 37°C in a humidified atmosphere containing 5% CO_2_.

### 2.8 Cell viability assay

After 24 h of co-culture, cell viability was assessed using the Cell Counting Kit-8 (CCK-8). Each well received 10 μL of CCK-8 reagent and was incubated at 37°C for 2 h. Absorbance was measured at 450 nm using a microplate reader to quantify cell viability.

## 3 Results

### 3.1 TIM-3 expression-based survival analysis, binding pocket characterization, and virtual screening

The identification of prognostic biomarkers and therapeutic targets is essential for improving clinical outcomes in cancer patients. In recent studies, TIM-3 has emerged as a promising candidate due to its potential role in tumor progression and immune regulation. Therefore, this study sought to evaluate the prognostic significance and druggability of TIM-3 by examining survival outcomes and performing structural analyses.

**FIGURE 1:**
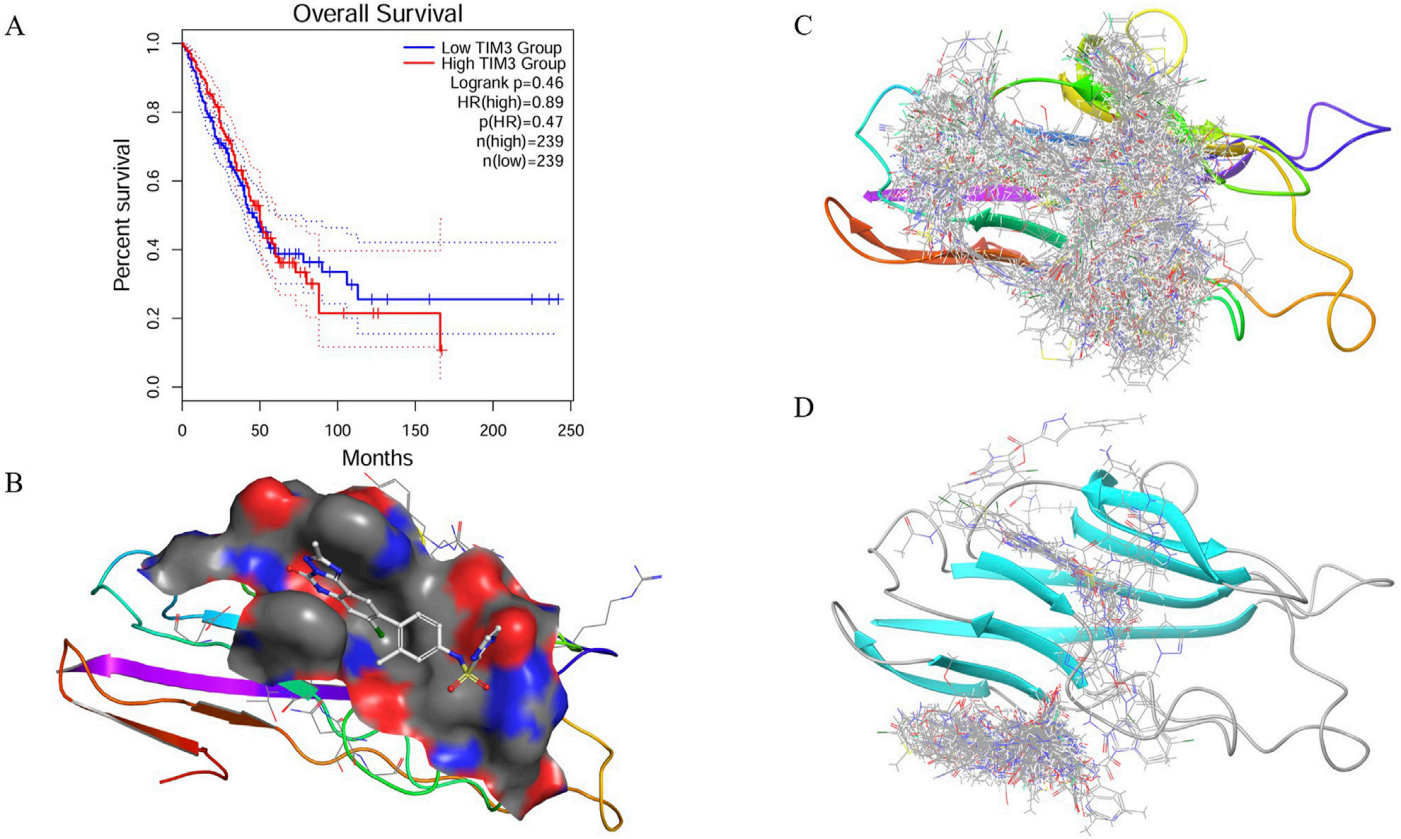
Prognostic relevance, pocket characterization, and virtual screening of TIM-3. **(A)** Kaplan–Meier survival analysis comparing overall survival between patients with high and low TIM-3 expression levels, revealing significant prognostic differences. **(B)** Structural analysis of the TIM-3 binding site using SiteMap, identifying a well-defined druggable pocket composed of charged, polar, hydrophobic, aromatic, and cysteine residues. **(C)** Virtual screening workflow employing a hierarchical docking strategy to identify potential high-affinity ligands from a large compound library. **(D)** Global docking validation of top-scoring compounds, confirming selective binding within the predicted TIM-3 pocket.

The survival analysis reveals a clear divergence in survival probabilities between patients with low and high TIM-3 expression, particularly notable after 50 months (Figure 1A). Patients exhibiting high TIM-3 expression levels demonstrated significantly poorer survival outcomes compared to those with low expression, underscoring the prognostic relevance of TIM-3 as a potential biomarker for adverse outcomes in the long term.

The binding pocket of TIM-3 was systematically characterized using the SiteMap tool, uncovering a druggable cavity with a surface area of 194.75 Å^2^ and a volume of 247.25 Å^3^ (Figure 1B). The pocket dimensions measured approximately 13.0 × 11.0 × 9.0 Å, with its center located at coordinates (−22.0, −27.0, 21.0) Å. Analysis of the pocket composition revealed a diverse environment consisting of charged, polar, hydrophobic, aromatic, and cysteine residues. Specifically, negatively charged residues such as GLU-2 and GLU-4 provide hydrogen bond acceptor sites, facilitating electrostatic interactions. Positively charged residues including LYS-101 and LYS-105 may participate in hydrogen bonding and salt bridge formation. Polar residues like ASN-103 and SER-1 contribute additional hydrogen bonding potential, enhancing ligand affinity. The hydrophobic core formed by residues VAL-3, LEU-104, ILE-86, ALA-36, PRO-16, and PRO-38 aids ligand stabilization. Additionally, aromatic residues TYR-5, TYR-87, PHE-18, and PHE-102 enable π-π stacking interactions, essential for binding aromatic ligands. Cysteine residues such as CYS-17, CYS-37, and CYS-89 may stabilize the structural integrity through disulfide bonds or serve as potential covalent interaction sites. The complex interplay of these residues suggests this binding pocket can accommodate ligands possessing diverse chemical functionalities to optimize binding affinity and stability.

The virtual screening utilized the TargetMol compound library containing 6.43 million compounds, employing a hierarchical screening approach through High-Throughput Virtual Screening (HTVS), Standard Precision (SP), and Extra Precision (XP) docking methods. Initial screening identified multiple compounds demonstrating strong predicted binding affinities, as indicated by low docking scores (Figure 1C). Notably, compounds HIT103355798 (−13.306), HIT101646735 (−13.294), HIT101475714 (−13.256), HIT105928298 (−13.250), and HIT102158461 (−13.128) exhibited the most favorable docking scores, suggesting robust interactions within the TIM-3 binding pocket. These initial docking results highlight a set of promising lead compounds warranting further exploration due to their potential high affinity interactions.

To validate the specificity of compound binding within the predicted pocket, global docking was performed on compounds with docking scores superior to the reference ligand N-(4-(8-chloro-2-methyl-5-oxo-5,6-dihydro-[1,2,4]triazolo [1,5-c]quinazolin-9-yl)-3-methylphenyl)-1H-imidazole-2-sulfonamide (−9.987). This global docking analysis revealed that only fifteen compounds retained binding within the initially predicted binding pocket (Figure 1D). Consequently, subsequent analyses were focused exclusively on these fifteen compounds due to their demonstrated specificity and stability of binding to the TIM-3 pocket.

### 3.2 MMGBSA-based binding free energy and interaction analysis of candidate TIM-3 inhibitors

**FIGURE 2:**
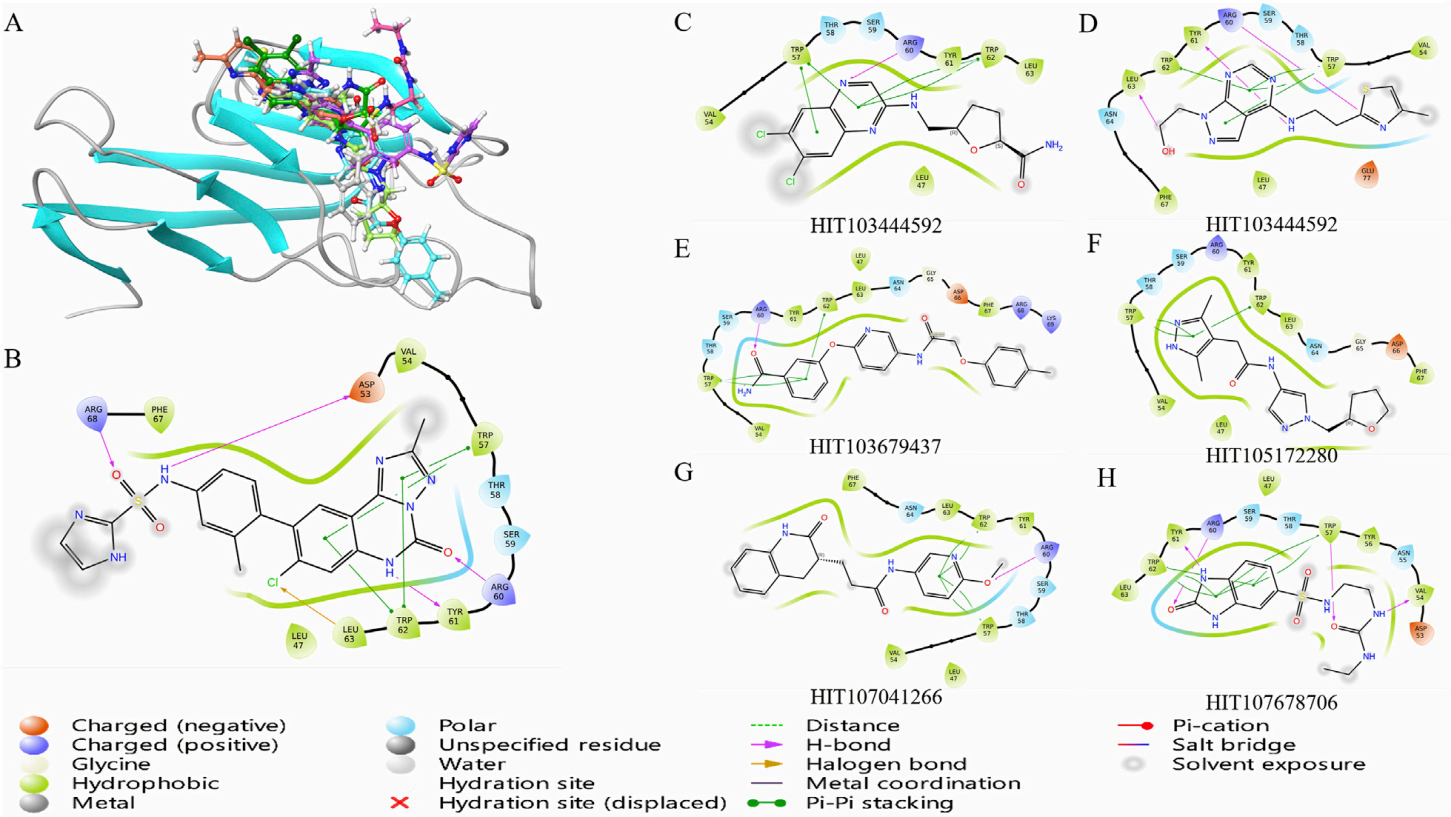
Binding free energy evaluation and interaction profiling of top TIM-3 ligands. **(A)** Binding free energy (MMGBSA ΔG_bind) of selected compounds and reference inhibitor within the TIM-3 binding site. Calculations were performed using Prime/MM-GBSA on Glide XP poses. **(B)** Comparative interaction profiling highlights the distinct binding modes of selected compounds relative to the reference ligand, focusing on key residues such as TRP-57 and ARG-60. **(C–H)** Molecular interaction diagrams illustrating detailed residue–ligand contacts for six candidate inhibitors, generated from the representative MD-stabilized conformations.

To further evaluate the binding potential of screened compounds, MMGBSA (Molecular Mechanics-Generalized Born Surface Area) binding free energy calculations were conducted, providing a detailed quantitative analysis of binding interactions (Figure 2A). Among these compounds, six demonstrated stronger predicted binding affinities compared to *N-(4-(8-chloro-2-methyl-5-oxo-5,6-dihydro-[1,2,4]triazolo* *[1,5-c]quinazolin-9-yl)-3-methylphenyl)-1H-imidazole-2-sulfonamide*, which exhibited an MMGBSA ΔG Bind score of −57.81 kcal/mol. HIT107678706 exhibited the strongest binding affinity with an MMGBSA score of −69.35 kcal/mol, closely followed by HIT104310526 (−67.16 kcal/mol) and HIT100058889 (−64.50 kcal/mol), highlighting significantly enhanced binding stability within the TIM-3 binding pocket. Detailed numerical data of the evaluated scores are summarized in Table 1.

**TABLE 1:** MMGBSA binding free energy values of selected compounds and the reference ligand against TIM-3.

Title	Docking score	MMGBSA dG Bind	Smiles
HIT107678706	−10.295	−69.35	CCNC(=O)NCCNS(=O) (=O)c1ccc2 [nH]c (=O)[nH]c2c1
HIT104310526	−10.588	−67.16	O=C(NCc1cccn2nccc12)c1cccc (-c2cn [nH]c2)c1
HIT100058889	−10.67	−64.5	NC(=O)[C@@H]1CC [C@H](CNc2cnc3cc (Cl)c (Cl)cc3n2)O1
HIT103679437	−10.253	−62.79	Cc1ccc (OCC(=O)Nc2ccc (Oc3cccc (C(N) = O)c3)nc2)cc1
HIT105172280	−10.765	−59.9	Cc1n [nH]c(C)c1CC(=O)Nc1cnn (C [C@H]2CCCO2)c1
HIT103444592	−10.336	−57.99	Cc1csc (CCNc2ncnc3c2cnn3CCO)n1
*N-(4-(8-chloro-2-methyl-5-oxo-5,6-dihydro-[1,2,4]triazolo[1,5-c]quinazolin-9-yl)-3-methylphenyl)-1H-imidazole-2-sulfonamide*	−9.987	−57.81	Cc1nc2c3cc (-c4ccc (NS(=O) (=O)c5ncc [nH]5)cc4C)c (Cl)cc3 [nH]c (=O)n2n1

The observed superior binding affinities are largely attributed to specific functional groups within these compounds. HIT107678706 contains amide and sulfonyl groups conducive to strong hydrogen bonding interactions, complemented by a heterocyclic structure facilitating electronic stabilization. HIT104310526 features ether linkages enhancing polarity and hydrogen bonding potential, along with a fused heterocyclic framework favorable for π-π stacking interactions. HIT100058889 incorporates amide and ether functionalities supporting hydrogen bonding, and chlorine substituents likely enhancing hydrophobic interactions. HIT103679437 is characterized by multiple aromatic systems and an ether group, ideal for π-π stacking and hydrogen bonding interactions. Similarly, HIT105172280 and HIT103444592 both contain ether linkages and heterocyclic rings optimizing interactions through hydrogen bonds and van der Waals contacts.

Analysis of compound binding modes showed distinct interaction patterns relative to *N-(4-(8-chloro-2-methyl-5-oxo-5,6-dihydro-* *[1,2,4]triazolo[1,5-c]quinazolin-9-yl)-3-methylphenyl)-1H-imidazole-2-sulfonamide* (Figure 2B). Specifically, HIT107678706 maintained interactions primarily with TRP-57, ARG-60, TYR-61, and TRP-62 but lacked interactions with ASP-53, LEU-63, and ARG-68. HIT104310526 predominantly engaged TRP-57, TRP-62, and LEU-63, emphasizing crucial aromatic interactions with fewer electrostatic contacts. HIT100058889 exhibited comprehensive interactions with TRP-57, ARG-60, TYR-61, and TRP-62, demonstrating significant electrostatic and aromatic stabilization.

The consistent involvement of TRP-57 and TRP-62 across compounds underscores the essential role of π-π stacking interactions in ligand stabilization within TIM-3. Enhanced interactions with ARG-60 and TYR-61 observed in HIT107678706, HIT100058889, HIT103679437, and HIT103444592 further indicate significant electrostatic and hydrogen bonding contributions. Compounds with fewer electrostatic interactions, such as HIT104310526 and HIT105172280, might rely predominantly on hydrophobic interactions, influencing their overall binding stability differently.

The six compounds identified as meeting the threshold criteria, namely, HIT107678706, HIT104310526, HIT100058889, HIT103679437, HIT105172280, and HIT103444592, exhibit particularly favorable interaction profiles and binding affinities (Figure 2C). These results underscore their potential as lead compounds for further optimization and investigation in TIM-3-targeted drug development.

### 3.3 Physicochemical property evaluation of screened TIM-3 inhibitor candidates

To further assess the drug-like properties and potential oral bioavailability of the six identified compounds (HIT107678706, HIT104310526, HIT100058889, HIT103679437, HIT105172280, HIT103444592), a systematic analysis of their physicochemical properties was performed in comparison with *N-(4-(8-chloro-2-methyl-5-oxo-5,6-dihydro-[1,2,4]triazolo[1,5-c]quinazolin-9-yl)-3-methylphenyl)-1H-imidazole-2-sulfonamide*. Parameters evaluated included molecular weight, hydrogen bond donors and acceptors, molecular flexibility, lipophilicity (LogP), aqueous solubility (LogS), distribution coefficient (LogD7.4), topological polar surface area (TPSA), and acid-base dissociation constants (pKa). These parameters were assessed against established theoretical thresholds to predict their suitability for drug development. Detailed numerical data of the evaluated physicochemical properties are summarized in Table 2.

**TABLE 2:** Physicochemical properties of selected compounds and *N-(4-(8-chloro-2-methyl-5-oxo-5,6-dihydro-[1,2,4]triazolo[1,5-c]quinazolin-9-yl)-3-methylphenyl)-1H-imidazole-2-sulfonamide*.

	HIT107678706	HIT104310526	HIT100058889	HIT103679437	HIT105172280	HIT103444592	References
(MW)	327.1	317.13	340.05	377.14	303.17	304.11	469.07
Volume	294.467	323.068	301.202	385.253	301.709	285.196	419.761
Density	1.111	0.982	1.129	0.979	1.005	1.066	1.117
nHA	9	6	6	7	7	7	10
nHD	5	2	3	3	2	2	3
nRot	8	5	4	8	6	6	4
nRing	2	4	3	3	3	3	5
MaxRing	9	9	10	6	5	9	13
nHet	10	6	8	7	7	8	12
fChar	0	0	0	0	0	0	0
nRig	14	22	17	20	16	15	29
Flexibility	0.571	0.227	0.235	0.4	0.375	0.4	0.138
Stereo Centers	0	0	2	0	1	0	0
TPSA	135.95	75.08	90.13	103.54	84.83	88.75	137.9
logS	−2.727	−3.109	−4.379	−3.305	−2.033	−2.298	−4.527
logP	0.054	2.334	2.574	2.046	0.93	1.174	2.75
logD7.4	0.631	2.486	2.493	2.395	1.387	1.657	2.64
pka (Acid)	8.135	10.13	7.25	9.262	8.401	9.051	8.651
pka (Base)	3.827	4.213	4.826	4.811	4.902	4.828	5.259

All six test compounds demonstrated molecular weights ranging from 303.17 to 377.14, within the favorable range (100–600), indicating potential suitability for oral administration. The hydrogen bonding profiles were balanced, with hydrogen bond acceptors ranging from 6 to 9 and donors from 2 to 5, comparable to *N-(4-(8-chloro-2-methyl-5-oxo-5,6-dihydro-[1,2,4]triazolo[1,5-c]quinazolin-9-yl)-3-methylphenyl)-1H-imidazole-2-sulfonamide* (nHA = 10, nHD = 3). Molecular flexibility was assessed based on rotatable bonds (4–8) and ring systems (2–4), demonstrating acceptable flexibility and rigidity profiles conducive to optimal receptor binding and bioavailability.

Regarding lipophilicity (LogP), all compounds displayed values within the optimal range (0–3), indicating balanced lipid solubility suitable for membrane permeability. However, aqueous solubility (LogS) analysis revealed HIT100058889 exhibited relatively low solubility (−4.379), suggesting potential formulation challenges. Additionally, distribution coefficients (LogD7.4) were generally within ideal parameters (1–3), though HIT107678706 displayed lower values (0.631), which might influence its bioavailability at physiological pH.

Topological polar surface area (TPSA) values for all compounds ranged from 75.08 to 135.95 Å^2^, within an acceptable range for maintaining permeability and polarity balance. Acid-base dissociation constants (pKa) indicated variable ionization profiles under physiological conditions, with pKa (Acid) ranging from 7.25 to 10.13 and pKa (Base) from 3.827 to 4.902. This suggests varying degrees of membrane permeability and solubility in physiological environments.

Collectively, these physicochemical property analyses highlight the potential drug-likeness of the evaluated compounds. While their molecular weights, hydrogen bonding capabilities, flexibility, and lipophilicity profiles indicate promising drug candidate characteristics, optimization may be necessary for certain compounds like HIT100058889 and HIT107678706 to address solubility and bioavailability concerns. Further experimental validation, including solubility assays, permeability studies, and pharmacokinetic evaluations, is recommended to confirm their suitability for drug development.

### 3.4 Binding stability of TIM-3 inhibitors HIT104310526 and HIT100058889 assessed by binding pose metadynamics

**FIGURE 3:**
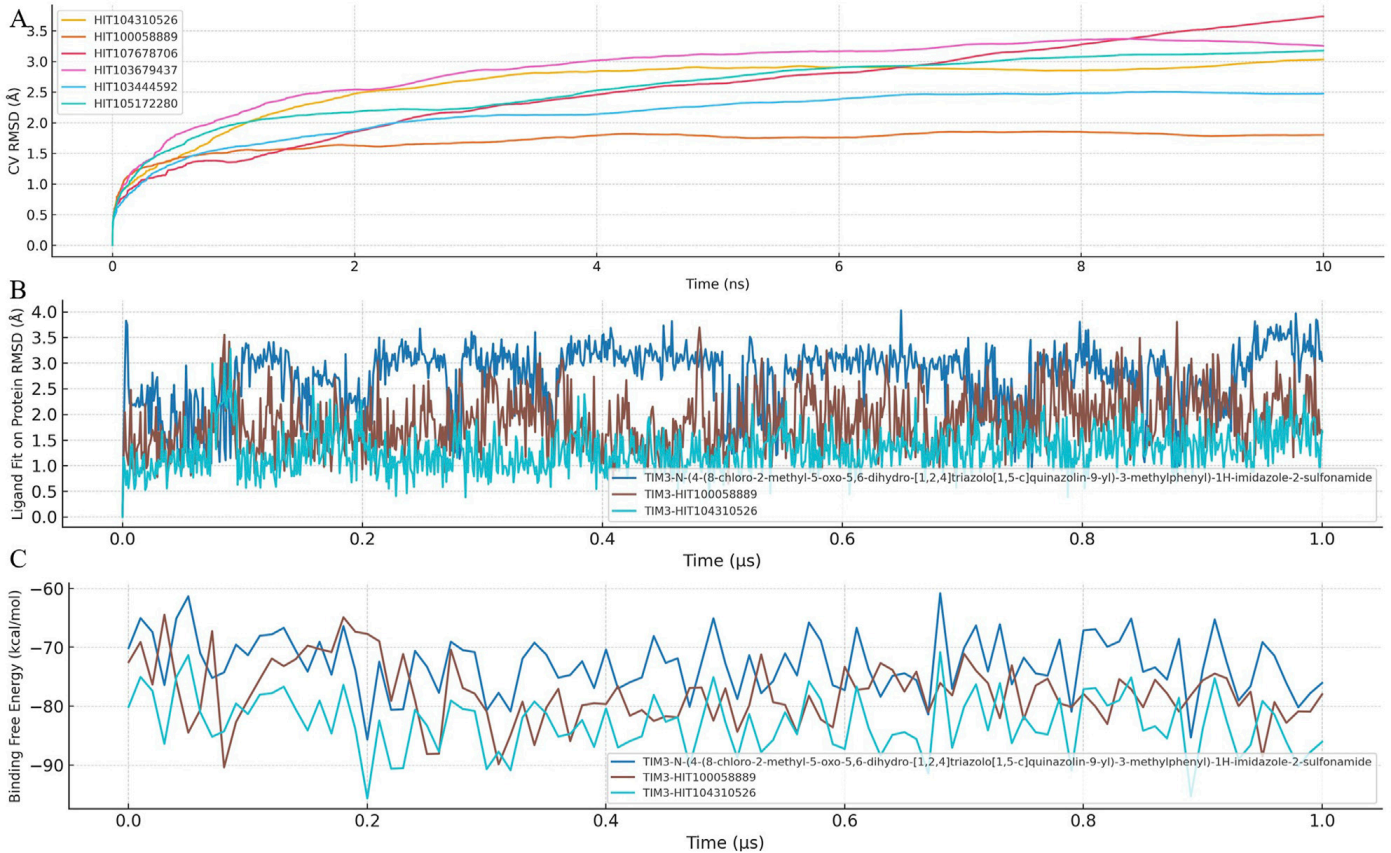
Binding stability and dynamic behavior of TIM-3 inhibitors assessed by BPMD and MD simulations. **(A)** Binding pose metadynamics (BPMD) simulations were conducted for HIT104310526, HIT100058889, and the reference compound to evaluate the dynamic stability of protein–ligand complexes. Each compound was subjected to ten independent 10 ns BPMD runs using ligand heavy atom RMSD as the collective variable (CV). Lower RMSD values and higher PersScore values indicate greater pose stability throughout the simulations. **(B)** Time-dependent RMSD trajectories of each ligand during conventional 1 μs molecular dynamics (MD) simulations, reflecting the conformational fluctuation and spatial retention of the ligands within the TIM-3 binding pocket. **(C)** Time-resolved MM-GBSA binding free energy profiles calculated across the entire 1 μs MD trajectory. Consistently lower and more stable ΔG_bind values reflect enhanced binding stability and affinity. Among all compounds, HIT104310526 showed the most stable pose and favorable binding energetics.

To evaluate the dynamic stability and robustness of ligand binding within the TIM-3 pocket, Binding Pose Metadynamics (BPMD) simulations were employed for selected compounds. BPMD simulations provide enhanced sampling to explore the conformational space of ligand-protein complexes, assessing how effectively compounds maintain their initial binding poses under simulated biological conditions. The dynamic fluctuations of CV RMSD over simulation time are illustrated in Figure 3A.

The BPMD analysis involved performing ten independent 10 ns simulations for each compound, using ligand heavy atom root-mean-square deviation (RMSD) as the collective variable (CV). Lower RMSD values throughout the simulation reflect stable binding poses, while higher RMSD values indicate potential displacement or weaker ligand binding stability.

Among the tested compounds, HIT104310526 exhibited remarkable stability, consistently maintaining minimal RMSD fluctuations, indicating a highly stable and well-defined binding orientation within TIM-3. Its superior stability was quantitatively supported by its Persistence Score (PersScore) of 1.000, indicating persistent occupancy of the initial binding pose throughout the simulation, and a Composite Score (CompScore) of −2.07, highlighting strong binding affinity and stability.

Similarly, HIT100058889 also demonstrated notable binding stability with relatively low RMSD fluctuations during the simulations, achieving a PersScore of 0.677 and a CompScore of −1.577, further validating its robust interaction with the TIM-3 pocket.

Overall, these BPMD results highlight HIT104310526 and HIT100058889 as highly promising candidates due to their superior binding stability profiles. Future investigations should prioritize the optimization of these compounds, emphasizing improvements in pharmacokinetic properties while retaining their demonstrated stability and affinity within the TIM-3 binding site.

### 3.5 Integrated MD simulation and MM-GBSA analysis reveals superior stability of HIT104310526 in TIM-3 binding

To further investigate ligand-protein interaction dynamics, a comprehensive analysis combining ligand fit on protein RMSD and MM-GBSA binding free energy was conducted. This integrated approach provided detailed insights into the stability and binding affinity of selected compounds within the TIM-3 binding pocket. The temporal dynamics of ligand RMSD and MM-GBSA binding free energy are presented in Figures 3B,C, respectively.

Analysis of RMSD trajectories revealed significant variability in ligand stability across the tested compounds. *N-(4-(8-chloro-2-methyl-5-oxo-5,6-dihydro-[1,2,4]triazolo[1,5-c]quinazolin-9-yl)-3-methylphenyl)-1H-imidazole-2-sulfonamide* exhibited notable RMSD fluctuations, indicating instability and potential shifts in its binding pose. In contrast, HIT100058889 showed moderate fluctuations during initial simulation stages but subsequently stabilized, suggesting robust binding interactions. HIT104310526 exhibited minimal RMSD deviations throughout the simulation period, indicating the highest degree of binding stability among the analyzed compounds.

Binding free energy calculations provided complementary evidence of compound stability and affinity. *N-(4-(8-chloro-2-methyl-5-oxo-5,6-dihydro-[1,2,4]triazolo[1,5-c]quinazolin-9-yl)-3-methylphenyl)-1H-imidazole-2-sulfonamide* presented an average MM-GBSA binding free energy of −72.87 kcal/mol, whereas HIT100058889 demonstrated stronger binding with a more negative value of −77.84 kcal/mol. HIT104310526 showed the most favorable binding affinity, exhibiting the lowest binding free energy (−82.87 kcal/mol) with minimal fluctuations, confirming its superior binding stability and affinity.

Collectively, these findings underscore HIT104310526 as the most promising candidate, characterized by exceptional binding stability and high affinity to TIM-3. HIT100058889 also emerges as a favorable compound warranting further consideration. In contrast, the substantial conformational shifts observed with *N-(4-(8-chloro-2-methyl-5-oxo-5,6-dihydro-[1,2,4]triazolo[1,5-c]quinazolin-9-yl)-3-methylphenyl)-1H-imidazole-2-sulfonamide* highlight potential limitations in its binding stability. Future experimental validation and optimization of HIT104310526 and HIT100058889 are recommended to substantiate their therapeutic potential.

### 3.6 Residue flexibility and structural rearrangements of TIM-3 induced by HIT104310526 and HIT100058889

**FIGURE 4:**
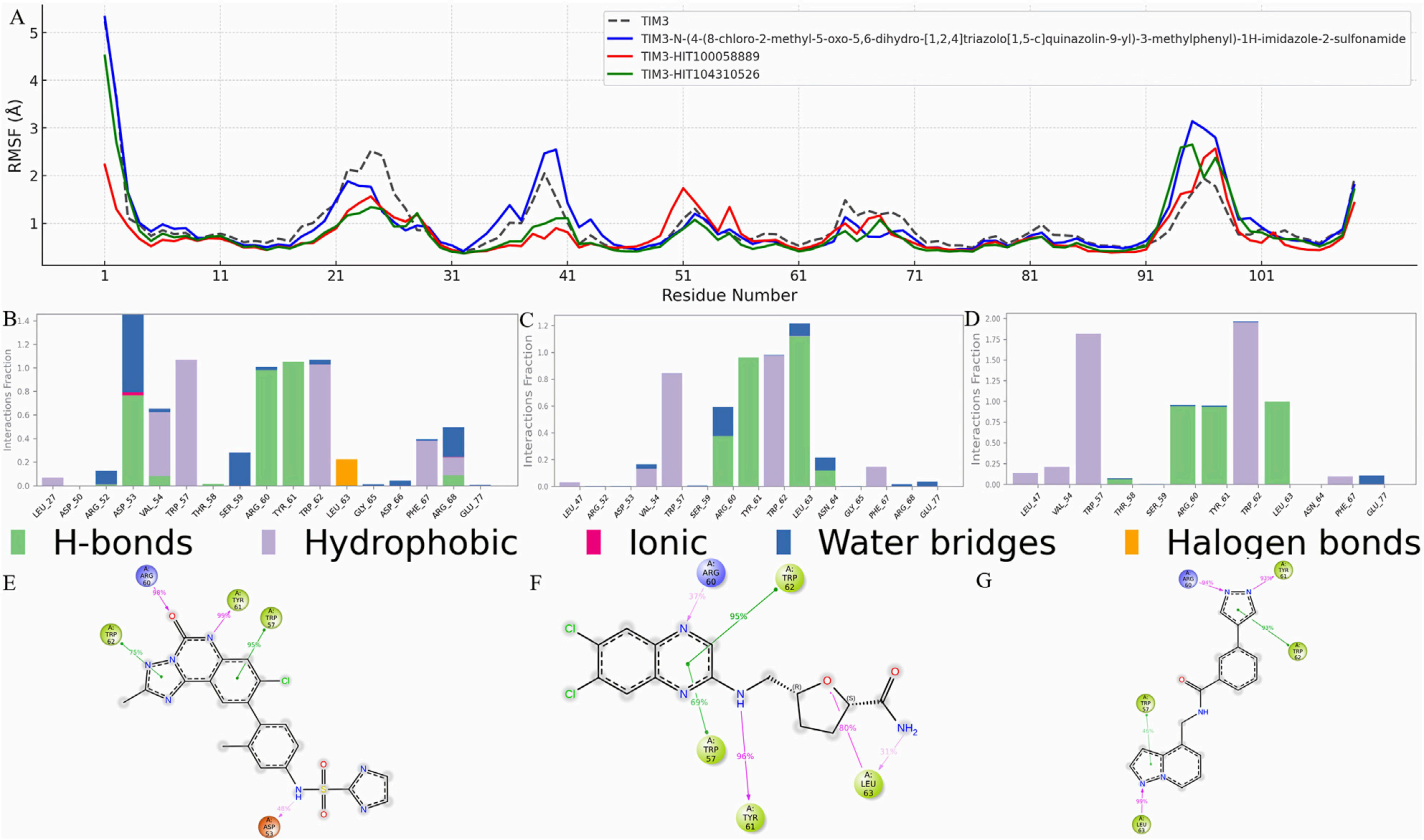
Residue flexibility and interaction profile of TIM-3 in response to ligand binding. **(A)** RMSF analysis of TIM-3 backbone atoms to assess residue-level flexibility changes upon binding with different ligands. Notable fluctuations were observed in specific regions, indicating structural adaptations induced by ligand interactions. **(B–D)** Interaction frequency profiles for the reference compound, HIT100058889, and HIT104310526, respectively, highlighting key residues contributing to stable binding within the TIM-3 pocket. **(E–G)** Schematic representations of ligand–residue contacts observed over the course of the molecular dynamics simulations, detailing atom-level interactions that govern binding stability and specificity for each compound.

To elucidate the structural impact of ligand binding on TIM-3, residue flexibility was evaluated through Root Mean Square Fluctuation (RMSF) analysis. Significant RMSF variations were predominantly observed in regions spanning residues 15-60 and 91-101, which likely reflect structural adjustments induced by ligand interactions (Figure 4A).

Residues such as GLY-25 and PRO-24 exhibited substantial decreases in RMSF values across all analyzed compounds, including *N-(4-(8-chloro-2-methyl-5-oxo-5,6-dihydro-[1,2,4]triazolo[1,5-c]quinazolin-9-yl)-3-methylphenyl)-1H-imidazole-2-sulfonamide*, HIT100058889, and HIT104310526, indicating these residues form part of the core binding pocket stabilized upon ligand interaction. Additionally, specific residues such as ASN-26 and LEU-27 showed reduced flexibility exclusively in the presence of *N-(4-(8-chloro-2-methyl-5-oxo-5,6-dihydro-[1,2,4]triazolo[1,5-c]quinazolin-9-yl)-3-methylphenyl)-1H-imidazole-2-sulfonamide*, whereas VAL-39 demonstrated flexibility reduction uniquely upon binding with HIT100058889 and HIT104310526.

In contrast, residues GLY-95 and PRO-94 displayed increased flexibility following interactions with *N-(4-(8-chloro-2-methyl-5-oxo-5,6-dihydro-[1,2,4]triazolo[1,5-c]quinazolin-9-yl)-3-methylphenyl)-1H-imidazole-2-sulfonamide* and HIT104310526. Similarly, residues ILE-96 and MET-97 exhibited RMSF increases with all tested compounds, indicating dynamic structural alterations at these distal regions. HIT100058889 notably influenced residues GLU-51, ASN-55, and ASP-50, enhancing their flexibility in a distinct modulation pattern compared to other tested compounds.

Detailed interaction analyses further validated these observations. Interaction frequency profiles for *N-(4-(8-chloro-2-methyl-5-oxo-5,6-dihydro-[1,2,4]triazolo[1,5-c]quinazolin-9-yl)-3-methylphenyl)-1H-imidazole-2-sulfonamide*, HIT100058889, and HIT104310526 are depicted in Figures 4B–D, highlighting crucial residues contributing significantly to binding stability. Furthermore, detailed ligand-atom interactions with specific protein residues are schematically illustrated in Figures 4E–G, providing deeper insights into residue-specific binding mechanisms and interaction dynamics.

Collectively, these RMSF and interaction analyses underscore key residues implicated in ligand-induced stability changes and structural adaptations, facilitating a comprehensive understanding of ligand-protein interaction dynamics crucial for drug design and optimization targeting TIM-3.

### 3.7 Comparative toxicity evaluation of HIT104310526, HIT100058889, and *N-(4-(8-chloro-2-methyl-5-oxo-5,6-dihydro-[1,2,4]triazolo[1,5-c]quinazolin-9-yl)-3-methylphenyl)-1H-imidazole-2-sulfonamide*

To ensure the safety profile of potential therapeutic candidates, a comprehensive comparative toxicity analysis was conducted for HIT100058889, HIT104310526, and *N-(4-(8-chloro-2-methyl-5-oxo-5,6-dihydro-[1,2,4]triazolo[1,5-c]quinazolin-9-yl)-3-methylphenyl)-1H-imidazole-2-sulfonamide*. This analysis included assessments across 24 different toxicity endpoints (Table 3).

**TABLE 3:** Comparative toxicity profiling of HIT100058889, HIT104310526, and the reference compound across 24 toxicity endpoints.

	N-(4-(8-chloro-2-methyl-5-oxo-5,6-dihydro-[1,2,4]triazolo [1,5-c]quinazolin-9-yl) -3-methylphenyl)-1H-imidazole-2-sulfonamide	HIT100058889	HIT104310526
hERG Blockers	0.124	0.608	0.651
hERG Blockers (10um)	0.169	0.643	0.499
DILI	1	0.992	0.986
AMES Toxicity	0.534	0.905	0.761
Rat Oral Acute Toxicity	0.577	0.666	0.555
FDAMDD	0.76	0.892	0.571
Skin Sensitization	0.076	0.986	0.026
Carcinogenicity	0.822	0.832	0.807
Eye Corrosion	0	0	0
Eye Irritation	0.109	0.245	0.107
Respiratory	0.449	0.547	0.492
Human Hepatotoxicity	0.971	0.803	0.829
Drug-induced Nephrotoxicity	0.707	0.941	0.873
Drug-induced Neurotoxicity	0.683	0.975	0.877
Ototoxicity	0.87	0.721	0.615
Hematotoxicity	0.66	0.535	0.38
Genotoxicity	1	0.999	0.999
RPMI-8226 Immunitoxicity	0.05	0.183	0.14
A549 Cytotoxicity	0.034	0.177	0.84
Hek293 Cytotoxicity	0.168	0.801	0.549
BCF	1.139	0.821	0.73
IGC50	3.947	3.555	3.459
LC50DM	4.916	4.888	4.902
LC50FM	4.587	4.255	4.315

Both HIT100058889 and HIT104310526 demonstrated higher scores in hERG blocking assays (including evaluations at a concentration of 10 µM) compared to *N-(4-(8-chloro-2-methyl-5-oxo-5,6-dihydro-[1,2,4]triazolo[1,5-c]quinazolin-9-yl)-3-methylphenyl)-1H-imidazole-2-sulfonamide*, suggesting potentially enhanced cardiotoxic risk. Specifically, HIT100058889 exceeded by scores of 0.484 and 0.474 at standard and 10 µM concentrations, respectively, while HIT104310526 displayed elevated scores by 0.527 and 0.330 in these assays.

In Drug-Induced Liver Injury (DILI) assessments, both compounds exhibited scores slightly lower than those of *N-(4-(8-chloro-2-methyl-5-oxo-5,6-dihydro-[1,2,4]triazolo[1,5-c]quinazolin-9-yl)-3-methylphenyl)-1H-imidazole-2-sulfonamide*, indicating comparable hepatic safety profiles. Additionally, both HIT100058889 and HIT104310526 performed better in AMES toxicity and A549 cytotoxicity assays, with HIT104310526 showing notably higher cytotoxic potential towards A549 cells, exceeding by 0.806 points.

Environmental toxicity indicators such as Bioconcentration Factor (BCF) and Growth Inhibition Concentration (IGC50) revealed generally lower scores for HIT100058889 and HIT104310526 compared to *N-(4-(8-chloro-2-methyl-5-oxo-5,6-dihydro-[1,2,4]triazolo[1,5-c]quinazolin-9-yl)-3-methylphenyl)-1H-imidazole-2-sulfonamide*, indicating a potentially lower environmental risk.

Overall, this comprehensive toxicity profiling highlights HIT100058889 and HIT104310526 as viable therapeutic candidates with acceptable toxicity profiles. However, careful monitoring and targeted optimization are advised to mitigate identified cardiotoxic risks, particularly associated with hERG inhibition.

### 3.8 Selective cytotoxicity of HIT104310526 and HIT100058889 against NSCLC (A549) and normal (BEAS-2) cell lines

To comprehensively evaluate the selective cytotoxicity of HIT100058889 and HIT104310526, cell viability assays were performed using the A549 non-small cell lung cancer (NSCLC) cell line and the normal bronchial epithelial cell line BEAS-2. The dose-dependent cytotoxic effects were assessed using the CCK-8 assay.

**FIGURE 5:**
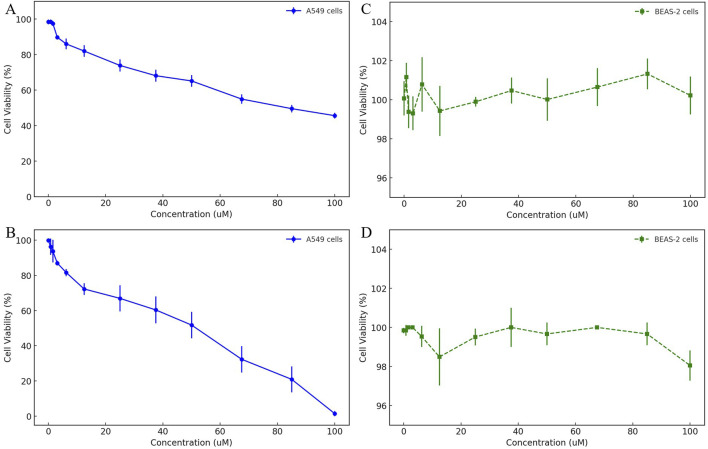
Dose-dependent cytotoxicity evaluation of HIT100058889 and HIT104310526 in cancerous and normal lung cell lines. **(A, B)** CCK-8 assay results depicting dose-dependent effects of HIT100058889 and HIT104310526 on the viability of A549 NSCLC cells. Each point represents the mean ± SD of three independent replicates. IC_50_ values were calculated using nonlinear regression. **(C, D)** Cytotoxicity assessment of the same compounds in BEAS-2B normal bronchial epithelial cells, showing no significant cytotoxicity across all tested concentrations.

In the A549 cell line, HIT100058889 exhibited relatively mild cytotoxic effects, maintaining cell viability above 60% between 12.5 μM and 50 μM, with approximately 45% viability observed at the highest concentration tested (100 μM). The calculated IC50 value was 91.82 μM, indicating moderate cytotoxic efficacy (Figure 5A). Conversely, HIT104310526 displayed significant dose-dependent inhibition, with minimal cytotoxicity observed below 6.25 μM. Substantial decreases in viability commenced at 12.5 μM, dropping below 20% at concentrations of 85 μM and 100 μM, resulting in a calculated IC50 value of approximately 37.74 μM, highlighting strong anticancer potential (Figure 5B).

In the normal bronchial epithelial cell line BEAS-2, both compounds demonstrated negligible cytotoxic effects. Specifically, HIT100058889 treatment maintained cell viability consistently above 95% across the entire concentration range tested (0–100 μM) (Figure 5C). Similarly, HIT104310526 treatment exhibited minimal cytotoxicity with cell viability also maintained above 95% across all tested concentrations (Figure 5D). No statistically significant differences were observed among treatment groups.

These findings underscore the tumor-selective cytotoxic profiles of both HIT100058889 and HIT104310526, highlighting their potential therapeutic value with an advantageous safety profile. Further mechanistic investigations into apoptosis induction and cell cycle arrest are warranted to elucidate the underlying cytotoxic mechanisms of these compounds.

## 4 Discussion

HIT104310526 (*N-[(1H-1,2,4-triazol-3-yl)phenyl]-1-(1H-pyrazolo[3,4-b]pyridin-3-yl)methanamide*) was identified as a potent and selective TIM-3 inhibitor through a synergistic combination of *in silico* and *in vitro* approaches. Mechanistically, our findings suggest that HIT104310526 binds deeply within the TIM-3 IgV-domain pocket–the same interface that TIM-3 uses to engage its ligands–thereby blocking critical TIM-3–ligand interactions (Chongsaritsinsuk et al., 2023; Gandhi et al., 2018). In molecular docking and dynamics simulations, HIT104310526 formed stable π–π stacking interactions with aromatic residues (notably TRP-57 and TRP-62) and hydrogen bonds/salt bridges with key polar residues (such as ARG-60 and TYR-61) in the TIM-3 binding site. These interactions mirror those observed for high-affinity TIM-3 ligands, *N-(4-(8-chloro-2-methyl-5-oxo-5,6-dihydro-[1,2,4]triazolo[1,5-c]quinazolin-9-yl)-3-methylphenyl)-1H-imidazole-2-sulfonamide*, emphasizing the compound’s ability to anchor in the binding cleft.

The stability of HIT104310526s binding was further corroborated by molecular dynamics and binding pose metadynamics analyses: the ligand maintained a low RMSD and a persistent binding pose over extended simulations (Haloi et al., 2025), along with a highly favorable MMGBSA binding free energy relative to reference inhibitors (Wang et al., 2019). This superior stability and affinity indicate that HIT104310526 effectively “locks” TIM-3 in an inactive conformation, which would prevent TIM-3 from transmitting its immunosuppressive signal. Consistent with this mode of action, HIT104310526 demonstrated selective cytotoxicity toward NSCLC cells (A549) while sparing normal bronchial epithelial cells, suggesting it can exert anti-tumor effects without off-target toxicity to healthy cells (Feng et al., 2015; Sato et al., 2020). Specifically, HIT104310526 showed a sub-50 μM IC_50_ in A549 cells with minimal impact on BEAS-2 cell viability at equivalent doses–an encouraging therapeutic window that highlights its tumor-selective action. In contrast, the analogue HIT100058889 was markedly less potent.

In the broader context of immune checkpoint therapy, HIT104310526 offers a small-molecule strategy distinct from the monoclonal antibodies currently under clinical investigation for TIM-3. TIM-3 has emerged as an important target due to its role in T cell exhaustion and adaptive resistance to PD-1/PD-L1 blockade (Chen et al., 2024). For example, in anti-PD-1 refractory lung tumors, upregulation of TIM-3 on T cells drives immune escape, and adding a TIM-3-blocking antibody can restore anti-tumor immunity (Koyama et al., 2016). Early-phase trials with TIM-3 antibodies like cobolimab and sabatolimab have demonstrated that targeting TIM-3 is clinically feasible (Gomes de Morais et al., 2022). Cobolimab combined with a PD-1 inhibitor showed acceptable safety and modest efficacy in advanced NSCLC patients (Moreno et al., 2022). However, TIM-3 monotherapy has so far yielded limited responses, whereas dual blockade of TIM-3 and PD-1 produced better clinical outcomes.

These results underscore that TIM-3 inhibition is most effective as part of a combination strategy, likely due to TIM-3’s complementary role in sustaining tumor immune evasion (Dixon et al., 2021). HIT104310526 could offer advantages in this setting: as a small molecule, it may penetrate tissues and tumor microenvironments more readily than antibodies and could be administered orally, providing flexible dosing and potentially lower cost (Moreno et al., 2022). Moreover, its direct cytotoxic effect on NSCLC cells—distinct from immune-dependent mechanisms—represents a unique feature. This dual action may offer enhanced efficacy when used in combination with immune checkpoint inhibitors, targeting both immune evasion and tumor-intrinsic survival pathways.

Comparable small-molecule TIM-3 inhibitors, such as ML-T7 (Ma et al., 2023) and SMI402 (Wu et al., 2023), have shown preclinical success. ML-T7 binds the FG-CC′ cleft of TIM-3, disrupting ligand interactions and enhancing antitumor immunity, especially when combined with PD-1 blockade. SMI402 blocks multiple TIM-3 ligands and restores exhausted T cell function in murine tumor models. HIT104310526 is consistent with these findings in its ability to engage the TIM-3 ligand-binding pocket. However, its demonstrated selective toxicity toward NSCLC cells may offer an additional therapeutic advantage not observed in other reported inhibitors. Whether this is due to TIM-3-dependent mechanisms in tumor cells or off-target effects requires further study.

Despite its potential, HIT104310526 exhibits limitations that warrant optimization. Predicted aqueous solubility was suboptimal, which may impair oral bioavailability (Bhalani et al., 2022). Future strategies to enhance solubility could involve introducing polar groups, designing prodrugs, or developing nanoformulations (Miller et al., 2018). Medicinal chemistry should aim to balance improved solubility with maintained potency and selectivity. Additionally, predictive toxicity profiling indicated elevated risk of hERG channel inhibition (Garrido et al., 2020). This represents a common cardiotoxicity risk and a frequent cause of clinical failure. Structural refinements aimed at reducing aromaticity, modifying pKa, or adjusting lipophilicity may help reduce this liability (Rokitskaya et al., 2021).

While our *in silico* analysis did not suggest major hepatotoxicity or genotoxicity, further validation using *in vitro* cardiomyocyte assays and *in vivo* toxicology studies will be necessary. Moreover, although the compound showed no strong liabilities in CYP450 metabolism predictions, experimental confirmation in human liver microsomes and pharmacokinetic profiling are needed. HIT104310526 therefore represents a strong hit compound that requires iterative optimization to refine its drug-likeness, especially concerning solubility and cardiotoxicity. Although our data support TIM-3 engagement as a key mechanism, we cannot rule out the possibility that HIT104310526 exerts additional cytotoxic effects through TIM-3-independent pathways. Prior studies have shown that small-molecule TIM-3 inhibitors may influence tumor-intrinsic survival pathways, such as PI3K/AKT or MAPK signaling (Ma et al., 2023; Wu et al., 2023). Moreover, our assays were conducted in cancer cell monocultures, which lack the immune cellular context necessary to confirm immune checkpoint-specific effects. Further validation using genetic knockdown of TIM-3 or rescue experiments in TIM-3-negative cells will be necessary to definitively establish the compound’s on-target specificity. These studies will help delineate whether the observed selective cytotoxicity results exclusively from TIM-3 blockade or from parallel signaling interference.

In addition to solubility and metabolic liabilities, cardiotoxicity due to predicted hERG channel inhibition represents another major concern for HIT104310526. hERG blockade is a frequent cause of attrition in drug development and is typically associated with structural features such as high lipophilicity, basic amines, and planar aromatic systems (Garrido et al., 2020). As part of future lead optimization, several strategies can be considered to mitigate hERG binding risk: (i) reducing the overall LogP of the molecule; (ii) disrupting planar aromatic moieties that facilitate π–π stacking with the hERG pore; and (iii) incorporating polar or sterically hindered substituents to reduce membrane permeability and binding affinity to cardiac ion channels. For HIT104310526, medicinal chemistry efforts may focus on modifying peripheral heterocycles or introducing solubilizing side chains that simultaneously address aqueous solubility and cardiotoxicity. Such structural refinements are essential to transition this hit compound into a viable clinical candidate with an acceptable safety profile.

Lastly, this study demonstrates the value of an integrated computational–experimental workflow for accelerating drug discovery. Structure-based virtual screening efficiently narrowed down millions of compounds to high-scoring candidates. Molecular dynamics and metadynamics simulations elucidated binding behavior at atomic resolution, identifying HIT104310526s favorable interaction network. Experimental assays subsequently validated its biological activity and selectivity. This closed-loop strategy ensured that computational predictions translated into meaningful biological effects, reducing development time and increasing discovery efficiency.

The successful identification and validation of HIT104310526 involved not only computational modeling but also survival analysis, physicochemical evaluation, toxicity profiling, and cellular assays. Each layer of analysis provided critical evidence for the compound’s potential as a TIM-3-targeted therapeutic. In the context of NSCLC and broader cancer immunotherapy, such integrative approaches are essential to navigate complex immunological pathways and identify candidates with both mechanistic and translational relevance.

Whether this selective effect is entirely TIM-3-dependent or involves off-target mechanisms remains to be elucidated. Previous studies have demonstrated that TIM-3 is not only expressed on immune cells but also aberrantly expressed on NSCLC tumor cells, including A549. For example, Gao et al. observed TIM-3 positivity in both NSCLC tumor tissues and the A549 cell membrane (Gao et al., 2012). More recently, Chen et al. reported that TIM-3 functions as a pan-cellular immunosuppressive molecule that contributes to both T cell exhaustion and tumor-intrinsic resistance to immune checkpoint therapy (Chen et al., 2024). The observed selective cytotoxicity of HIT104310526 toward A549 but not BEAS-2B cells may therefore reflect, at least in part, direct TIM-3 engagement on tumor cells. Nevertheless, we acknowledge the need for further validation—such as flow cytometry or immunostaining of TIM-3 in A549 cells—to definitively confirm target-specific effects and exclude potential off-target contributions.

## 5 Conclusion

In this study, HIT104310526 was identified as a potent and selective TIM-3 inhibitor through a comprehensive computational and experimental strategy. The compound exhibited high binding affinity and conformational stability within the TIM-3 pocket, as well as selective cytotoxicity toward NSCLC cells with minimal effects on normal epithelial cells. These findings highlight the compound’s dual potential to modulate immune checkpoints and directly inhibit tumor cell proliferation. Despite favorable activity profiles, limitations in aqueous solubility and predicted cardiotoxicity highlight the need for further lead optimization. Overall, HIT104310526 represents a promising candidate for NSCLC immunotherapy, supporting future development through structural refinement, preclinical validation, and potential combination strategies with existing immune checkpoint inhibitors.

## Data Availability

The raw data supporting the conclusions of this article will be made available by the authors, without undue reservation.
